# Mathematical Study and Numerical Simulation of Multispectral Bioluminescence Tomography

**DOI:** 10.1155/IJBI/2006/54390

**Published:** 2006-12-12

**Authors:** Weimin Han, Wenxiang Cong, Ge Wang

**Affiliations:** ^1^ Department of Mathematics, University of Iowa, Iowa City, IA 52242, USA; ^2^ Division of Biomedical Imaging, VT-WFU School of Biomedical Engineering and Sciences, Virginia Polytechnic Institute and State University, Blacksburg, VA 24061, USA

## Abstract

Multispectral bioluminescence tomography (BLT) attracts increasingly more attention in the area of optical molecular imaging. In this paper, we analyze the properties of the solutions to the regularized and discretized multispectral BLT problems. First, we show the solution existence, uniqueness, and its continuous dependence on the data. Then, we introduce stable numerical schemes and derive error estimates for numerical solutions. We report some numerical results to illustrate the performance of the numerical methods on the quality of multispectral BLT reconstruction.


Hindawi Publishing Corporation
International Journal of Biomedical Imaging
Volume 2006, Article ID 54390, Pages 1–10
DOI 10.1155/IJBI/2006/54390

### Mathematical Study and Numerical Simulation of Multispectral Bioluminescence Tomography

**Weimin Han,[1] Wenxiang Cong,[2] and Ge Wang[2]**

[1] *Department of Mathematics, University of Iowa, Iowa City, IA 52242, USA*
[2] *Division of Biomedical Imaging, VT-WFU School of Biomedical Engineering and Sciences, Virginia Polytechnic Institute and State University, Blacksburg, VA 24061, USA*

Received 8 April 2006; Revised 7 August 2006; Accepted 7 September 2006

Recommended for Publication by Ming Jiang

Multispectral bioluminescence tomography (BLT) attracts increasingly more attention in the area of optical molecular imaging. In this paper, we analyze the properties of the solutions to the regularized and discretized multispectral BLT problems. First, we show the solution existence, uniqueness, and its continuous dependence on the data. Then, we introduce stable numerical schemes and derive error estimates for numerical solutions. We report some numerical results to illustrate the performance of the numerical methods on the quality of multispectral BLT reconstruction.

Copyright © 2006 Weimin Han et al. This is an open access article distributed under the Creative Commons Attribution License, which permits unrestricted use, distribution, and reproduction in any medium, provided the original work is properly cited.

### 1. INTRODUCTION

Genetically engineered mice are popular laboratory models for numerous studies directly relevant to the human healthcare. Mouse imaging allows in vivo evaluation of physiological and pathological processes under controlled conditions simulating therapeutic interventions. One of the most promising mouse imaging modalities is based on luciferase report gene [1, 2]. When the corresponding substrate is administered into a mouse, the resultant biochemical reaction generates bioluminescent light at visible and infrared wavelengths. These bioluminescent photons carry important information about tumor burden, micro-metastases, infectious loci, therapeutic gene delivery, and so on. Bioluminescent imaging (BLI) utilizes sensitive photon detection techniques to observe bioluminescent signals on the body surface of the mouse.

Funded by NIH/NIBIB, we built the first bioluminescence tomography (BLT) prototype [3], and developed the BLT theory and methods [4–8]. Quickly, BLT has grown into a hot area, in which several groups are actively producing valuable results [9–14]. Currently, we are improving our BLT prototype with more features and better performance in hope that BLT will become a universal and powerful tool for molecular and cellular imaging in the near future. While multispectral BLT results were already reported in the literature [6, 12, 14], there is a critical need to establish a mathematical theory of multispectral BLT.

It is well known that all the common luciferase enzymes, from firefly (Fluc), click beetle (CBGr68, CBRed) and Renilla reniformins (HRLuc), share a wide spectral range, roughly from 400 to 750 nm [15]. Furthermore, the newly developed tricolor reporter generates bioluminescent light that is rich in green and red spectral bands. Because the optical properties of the tissues depend on the wavelength, the intensity and spectrum of bioluminescent light measured on the body surface of a mouse are a function of the spectrum of the bioluminescent source, its 3D distribution, and the individual anatomy of the mouse. Clearly, multispectral data are more informative than mixed data, and must be analyzed for the optimal BLT performance.

This paper provides a theoretical study of the multispectral BLT model. The spectrum is divided into certain numbers of bands, say $i_0$ bands $\Lambda_1, \ldots, \Lambda_{i_0}$, with $\Lambda_i = [\lambda_{i-1}, \lambda_i)$, $1 \le i \le i_0 - 1$, $\Lambda_{i_0} = [\lambda_{i_0-1}, \lambda_{i_0}]$. Here $\lambda_0 < \lambda_1 < \cdots < \lambda_{i_0}$ is a partition of the spectrum range. Denote by $p$ the bioluminescent source distribution function. Then the bioluminescent source distribution within the band $\Lambda_i$ is $\omega_i p$. In this paper, we always understand the range of the index $i$ to be

$\{1, \dots, i_0\}$. The weight $\omega_i \geq 0$, and $\sum_{i=1}^{i_0} \omega_i = 1$. Let $\Omega \subset \mathbb{R}^d$ ($d \leq 3$) be the biological medium with the boundary $\Gamma$. Let $\Omega_0$ be a measurable subset of $\Omega$ ($\Omega_0 = \Omega$ is allowed) where the light source exists. The set $\Omega_0$ is known as the permissible region. For each spectral band $\Lambda_i$, $1 \leq i \leq i_0$, we use the following diffusion equation to describe the photon density $u_i$ in $\Lambda_i$:

$$-\operatorname{div}(D_i \nabla u_i) + \mu_{a,i} u_i = \omega_i p \chi_{\Omega_0} \quad \text{in } \Omega. \tag{1}$$

Here, $D_i(\mathbf{x}) = 1/[3(\mu_{a,i}(\mathbf{x}) + \mu'_{s,i}(\mathbf{x}))]$, $\mu_{a,i}(\mathbf{x})$ and $\mu'_{s,i}(\mathbf{x})$ are the absorption coefficient and the reduced scattering coefficient within the band $\Lambda_i$, $\chi_{\Omega_0}$ is the characteristic function of $\Omega_0$, that is, its value is 1 in $\Omega_0$, and is 0 in $\Omega \backslash \Omega_0$. The bioluminescent imaging experiments are usually performed in a dark environment so that the natural boundary condition takes the form [16]

$$u_i + 2AD_i \frac{\partial u_i}{\partial \nu} = 0 \quad \text{on } \Gamma. \tag{2}$$

Here, $\partial/\partial\nu$ stands for the outward normal derivative,

$$\begin{aligned} A(\mathbf{x}) &= \frac{1 + R(\mathbf{x})}{1 - R(\mathbf{x})}, \\ R(\mathbf{x}) &\approx -1.4399\gamma^{-2} + 0.7099\gamma^{-1} + 0.6681 + 0.0636\gamma, \end{aligned} \tag{3}$$

with $\gamma$ being the refractive index of the medium. Let $\Gamma_0 \subset \Gamma$ with $\text{meas}(\Gamma_0) > 0$ be a part of the boundary where measurement takes place; $\Gamma_0 = \Gamma$ is allowed. With the emission filter of bandpass $\Lambda_i$, the measured quantity is the outgoing flux density [16]:

$$Q_i = -D_i \frac{\partial u_i}{\partial \nu} = \frac{1}{2A} u_i \quad \text{on } \Gamma_0. \tag{4}$$

The pointwise formulation of the BLT problem is to determine the source function from (1)–(4) for $1 \leq i \leq i_0$. As noted in [8], the pointwise formulation (1)–(4) for $1 \leq i \leq i_0$ is ill-posed: (1) in general, there are infinite many solutions; (2) when the form of the source function is specified, generally there are no solutions; (3) the source function does not depend continuously on the data. We study the multispectral BLT problem through Tikhonov regularization as follows.

Denote by $Q_{\text{ad}}$ the admissible set of the source functions. We assume $Q_{\text{ad}}$ is a closed convex subset of $L^2(\Omega_0)$. Examples include $Q_{\text{ad}} = L^2(\Omega_0)$, or the subset of $L^2(\Omega_0)$ of nonnegatively valued functions, or certain finite dimensional subspace or subset of $L^2(\Omega_0)$. For a weak formulation of the boundary value problem (1)–(2), we need the space $V = H^1(\Omega)$. For any $q \in L^2(\Omega_0)$, and any $i = 1, \dots, i_0$, define $u_i = u_i(q) \in V$ to be the solution of the problem

$$\begin{aligned} \int_\Omega (D_i \nabla u_i \cdot \nabla v + \mu_{a,i} u_i v)\, dx + \int_\Gamma \frac{1}{2A} u_i v\, ds \\ = \int_{\Omega_0} \omega_i q v\, dx \quad \forall v \in V. \end{aligned} \tag{5}$$

From the assumptions on the data, we can apply the well-known Lax-Milgram lemma [17, 18] to conclude that the solution $u_i(q)$ exists and is unique. We note that the mapping $q \mapsto u_i(q)$ is linear.

Choose weights $w_i > 0$, $1 \leq i \leq i_0$, with $\sum_{i=1}^{i_0} w_i = 1$. Define a Tikhonov regularization functional [19, 20]

$$J_\varepsilon(q) = \sum_{i=1}^{i_0} w_i \| u_i(q) - 2AQ_i \|^2_{L^2(\Gamma_0)} + \varepsilon \|q\|^2_{L^2(\Omega_0)}, \quad \varepsilon \geq 0. \tag{6}$$

In the definition of $J_\varepsilon(\cdot)$, other norms than $\|\cdot\|_{L^2(\Gamma)}$ and $\|\cdot\|_{L^2(\Omega_0)}$ may be used if it is required to do so based on practical considerations. In this paper, we use (6) for the definition of $J_\varepsilon(\cdot)$ that leads to easier implementation. The following formulas for Gâteaux derivatives of $J_\varepsilon(\cdot)$ hold:

$$\begin{aligned} J'_\varepsilon(p)q &= 2\sum_{i=1}^{i_0} w_i \big(u_i(p) - 2AQ_i, u_i(q)\big)_{L^2(\Gamma_0)} + 2\varepsilon(p, q)_{L^2(\Omega_0)}, \\ J''_\varepsilon(p)q^2 &= 2\sum_{i=1}^{i_0} w_i \|u_i(q)\|^2_{L^2(\Gamma_0)} + 2\varepsilon\|q\|^2_{L^2(\Omega_0)} \end{aligned} \tag{7}$$

for $p, q \in L^2(\Omega_0)$. Thus, $J_\varepsilon(\cdot)$ is strictly convex when $\varepsilon > 0$. We then introduce the following multispectral BLT problem.

*Problem 1.* Find $p_\varepsilon \in Q_{\text{ad}}$ such that $J_\varepsilon(p_\varepsilon) = \inf_{q \in Q_{\text{ad}}} J_\varepsilon(q)$.

This paper is on a study of the multispectral BLT Problem 1. In the next section, we focus on the solution existence, uniqueness, and continuous dependence on the data. In Section 3, we discuss numerical methods for the multispectral BLT reconstruction and derive error estimates for the numerical solutions. In Section 4, we include some numerical examples to show the performance of the numerical methods. We end the paper by a concluding remark summarizing the main contributions of the paper.

### 2. WELL-POSEDNESS

We first address the existence and uniqueness issue. For this purpose, we make some assumptions on the given data. We assume $\Omega \subset \mathbb{R}^d$ is a nonempty, open, bounded set with a Lipschitz boundary $\Gamma$, $A \in [A_l, A_u]$ a.e. in $\Omega$ for some constants $0 < A_l \leq A_u < \infty$. For $i = 1, \dots, i_0$, we assume $D_i \in L^\infty(\Omega)$, $D_i \geq D_0$ a.e. in $\Omega$ for some constant $D_0 > 0$, $\mu_{a,i} \in L^\infty(\Omega)$, $\mu_{a,i} \geq 0$ a.e. in $\Omega$, $Q_i \in L^2(\Gamma_0)$, $\omega_i \geq \omega_0 > 0$ for some positive constant $\omega_0$.

**Theorem 1.** *For any $\varepsilon > 0$, Problem 1 has a unique solution $p_\varepsilon \in Q_{\text{ad}}$, which is characterized by a variational inequality*

$$\begin{aligned} \sum_{i=1}^{i_0} w_i \big(u_i(p_\varepsilon) - 2AQ_i, u_i(q - p_\varepsilon)\big)_{L^2(\Gamma_0)} \\ + \varepsilon(p_\varepsilon, q - p_\varepsilon)_{L^2(\Omega_0)} \geq 0 \quad \forall q \in Q_{\text{ad}}. \end{aligned} \tag{8}$$

*If $Q_{\text{ad}} \subset L^2(\Omega_0)$ is a subspace, then the solution $p_\varepsilon \in Q_{\text{ad}}$ is characterized by a variational equation*

$$\sum_{i=1}^{i_0} w_i(u_i(p_\varepsilon) - 2AQ_i, u_i(q))_{L^2(\Gamma_0)} + \varepsilon(p_\varepsilon, q)_{L^2(\Omega_0)} = 0 \quad \forall q \in Q_{\text{ad}}. \tag{9}$$

*Proof.* For $\varepsilon > 0$, Problem 1 is a constrained optimization for a strictly convex objective functional over a closed convex set. Thus, it has a unique solution (see, e.g., [17, Theorem 3.3.12]), and the solution $p_\varepsilon$ is characterized by the relation $J'_\varepsilon(p_\varepsilon)(q-p_\varepsilon) \geq 0$, for all $q \in Q_{\text{ad}}$ (see [17, Theorem 5.3.19]), which is the variational inequality (8). When $Q_{\text{ad}} \subset L^2(\Omega_0)$ is a subspace, the inequality (8) reduces to (9) by a standard argument. □

We then consider the continuous dependence of the solution on the data.

**Theorem 2.** *The solution $p_\varepsilon$ of Problem 1 depends continuously on the data.*

*Proof.* The solution $p_\varepsilon$ depends continuously on all the data. In order to maintain the length of the proof, in the following we show the continuous dependence of $p_\varepsilon$ on $\varepsilon$, $D_i$, $\mu_{a,i}$, $Q_i$ and $\omega_i$, $1 \leq i \leq i_0$. To simplify the notation, let $\overline{p}_\varepsilon \in Q_{\text{ad}}$ be the solution of Problem 1 with the data $\varepsilon+\delta_\varepsilon$ with $|\delta_\varepsilon| \leq \varepsilon/2$, and for $1 \leq i \leq i_0$, $Q_i + \delta_{Q_i} \in L^2(\Gamma_0)$, $D_i + \delta_{D_i} \in L^\infty(\Omega)$ with $\|\delta_{D_i}\|_{L^\infty(\Omega)} \leq D_0/2$, $\mu_{a,i}+\delta_{\mu_{a,i}} \in L^\infty(\Omega)$ with $\mu_{a,i}+\delta_{\mu_{a,i}} \geq 0$ a.e. in $\Omega$, and $\omega_i + \delta_{\omega_i}$ with $|\delta_{\omega_i}| \leq \omega_0/2$. Also, in this proof, we use $c$ for constants that may depend on $\varepsilon$, $D_0$, $\omega_0$, $Q_i$, and $\mu_{a,i}$ for $1 \leq i \leq i_0$, but are independent of $\delta_\varepsilon$, $\delta_{\omega_i}$, $\delta_{\mu_{a,i}}$, $\delta_{D_i}$, and $\delta_{Q_i}$ for $1 \leq i \leq i_0$.

Similar to (8), $\overline{p}_\varepsilon$ is characterized by the inequality

$$\sum_{i=1}^{i_0} w_i(\overline{u}_i(\overline{p}_\varepsilon) - 2A(Q_i + \delta_{Q_i}), \overline{u}_i(q - \overline{p}_\varepsilon))_{L^2(\Gamma_0)} + (\varepsilon + \delta_\varepsilon)(\overline{p}_\varepsilon, q - \overline{p}_\varepsilon)_{L^2(\Omega_0)} \geq 0 \quad \forall q \in Q_{\text{ad}}. \tag{10}$$

Take $q = p_\varepsilon$ in this inequality, $q = \overline{p}_\varepsilon$ in (8), and use these two inequalities to obtain

$$\begin{aligned}

&\sum_{i=1}^{i_0} w_i\|\overline{u}_i(\overline{p}_\varepsilon - p_\varepsilon)\|^2_{L^2(\Gamma_0)} + (\varepsilon + \delta_\varepsilon)\|\overline{p}_\varepsilon - p_\varepsilon\|^2_{L^2(\Omega_0)} \\

&\leq \sum_{i=1}^{i_0} w_i(u_i(p_\varepsilon) - 2AQ_i, \overline{u}_i(p_\varepsilon) - u_i(p_\varepsilon) + u_i(\overline{p}_\varepsilon) - \overline{u}_i(\overline{p}_\varepsilon))_{L^2(\Gamma_0)} \\

&\quad + \sum_{i=1}^{i_0} w_i(u_i(p_\varepsilon) - \overline{u}_i(p_\varepsilon), \overline{u}_i(\overline{p}_\varepsilon - p_\varepsilon))_{L^2(\Gamma_0)} \\

&\quad + \sum_{i=1}^{i_0} w_i(-2A\delta_{Q_i}, \overline{u}_i(p_\varepsilon - \overline{p}_\varepsilon))_{L^2(\Gamma_0)} \\

&\quad - \delta_\varepsilon(p_\varepsilon, \overline{p}_\varepsilon - p_\varepsilon)_{L^2(\Omega_0)}.

\end{aligned} \tag{11}$$

After some manipulations,

$$\begin{aligned}

&\max_{1\leq i\leq i_0} \|\overline{u}_i(\overline{p}_\varepsilon - p_\varepsilon)\|^2_{L^2(\Gamma_0)} + \|\overline{p}_\varepsilon - p_\varepsilon\|^2_{L^2(\Omega_0)} \\

&\leq c \max_{1\leq i\leq i_0} [\|\overline{u}_i(p_\varepsilon) - u_i(p_\varepsilon)\|_{L^2(\Gamma_0)} + \|\overline{u}_i(\overline{p}_\varepsilon) - u_i(\overline{p}_\varepsilon)\|_{L^2(\Gamma_0)}] \\

&\quad + c \max_{1\leq i\leq i_0} [\|\overline{u}_i(p_\varepsilon) - u_i(p_\varepsilon)\|^2_{L^2(\Gamma_0)} + \|\delta_{Q_i}\|^2_{L^2(\Gamma_0)}] + c|\delta_\varepsilon|^2.

\end{aligned} \tag{12}$$

By the definitions of $u_i(q)$ and $\overline{u}_i(q)$, we obtain the equality

$$\begin{aligned}

&\int_\Omega [(D_i + \delta_{D_i})\nabla(\overline{u}_i(q) - u_i(q)) \cdot \nabla v \\

&\quad + (\mu_{a,i} + \delta_{\mu_{a,i}})(\overline{u}_i(q) - u_i(q))v]dx \\

&\quad + \int_\Gamma \frac{1}{2A}(\overline{u}_i(q) - u_i(q))v\,ds \\

&= \int_{\Omega_0} \delta_{\omega_i} q v\,dx - \int_\Omega [\delta_{D_i}\nabla u_i(q) \cdot \nabla v + \delta_{\mu_{a,i}} u_i(q)v]dx

\end{aligned} \tag{13}$$

for any $v \in V$. Since $D_i + \delta_{D_i} \geq D_0/2 > 0$ and $\mu_{a,i} + \delta_{\mu_{a,i}} \geq 0$ a.e. in $\Omega$, we deduce that

$$\begin{aligned}

&\|\overline{u}_i(q) - u_i(q)\|_{H^1(\Omega)} \\

&\leq c[|\delta_{\omega_i}|\,\|q\|_{L^2(\Omega_0)} + \|\delta_{D_i}\|_{L^\infty(\Omega)}\|\nabla u_i(q)\|_{L^2(\Omega)^d} \\

&\quad + \|\delta_{\mu_{a,i}}\|_{L^\infty(\Omega)}\|u_i(q)\|_{L^2(\Omega)}].

\end{aligned} \tag{14}$$

Thus,

$$\begin{aligned}

&\max_{1\leq i\leq i_0} \|\overline{u}_i(p_\varepsilon) - u_i(p_\varepsilon)\|_{L^2(\Gamma_0)} \\

&\quad \leq c \max_{1\leq i\leq i_0} (|\delta_{\omega_i}| + \|\delta_{D_i}\|_{L^\infty(\Omega)} + \|\delta_{\mu_{a,i}}\|_{L^\infty(\Omega)}), \\

&\max_{1\leq i\leq i_0} \|\overline{u}_i(\overline{p}_\varepsilon) - u_i(\overline{p}_\varepsilon)\|_{L^2(\Gamma_0)} \\

&\quad \leq c \max_{1\leq i\leq i_0} (|\delta_{\omega_i}| + \|\delta_{D_i}\|_{L^\infty(\Omega)} + \|\delta_{\mu_{a,i}}\|_{L^\infty(\Omega)})\|\overline{p}_\varepsilon\|_{L^2(\Omega_0)}.

\end{aligned} \tag{15}$$

We can bound $\|\overline{p}_\varepsilon\|_{L^2(\Omega_0)}$ by $\|p_\varepsilon\|_{L^2(\Omega_0)}+\|\overline{p}_\varepsilon - p_\varepsilon\|_{L^2(\Omega_0)}$. Then from (12),

$$\begin{aligned}

&\max_{1\leq i\leq i_0} \|\overline{u}_i(\overline{p}_\varepsilon - p_\varepsilon)\|^2_{L^2(\Gamma_0)} + \|\overline{p}_\varepsilon - p_\varepsilon\|^2_{L^2(\Omega_0)} \\

&\quad \leq c\Big\{|\delta_\varepsilon|^2 + \max_{1\leq i\leq i_0} [|\delta_{\omega_i}| + \|\delta_{D_i}\|_{L^\infty(\Omega)} + \|\delta_{\mu_{a,i}}\|_{L^\infty(\Omega)}] \\

&\qquad + \max_{1\leq i\leq i_0} \|\delta_{Q_i}\|^2_{L^2(\Gamma_0)}\Big\}.

\end{aligned} \tag{16}$$

Hence, the solution depends continuously on the data. □

Next, we consider the solution behavior when $\varepsilon \to 0+$. Note that a solution $p \in Q_{\text{ad}}$ of Problem 1 with $\varepsilon = 0$ is characterized by the inequality

$$\sum_{i=1}^{i_0} w_i\big(u_i(p) - 2AQ_i, u_i(q-p)\big)_{L^2(\Gamma_0)} \geq 0 \quad \forall q \in Q_{\text{ad}}. \tag{17}$$

Let $S_0 \subset Q_{\text{ad}}$ be the solution set of Problem 1 with $\varepsilon = 0$. As in [21], the following result holds.

**Proposition 1.** *Assume $S_0$ is nonempty. Then $S_0$ is closed and convex. Moreover,*

$$p_\varepsilon \longrightarrow p_0 \quad \text{in } L^2(\Omega_0), \text{ as } \varepsilon \longrightarrow 0, \tag{18}$$

*where $p_0 \in S_0$ is the solution of Problem 1 with minimal $L^2(\Omega_0)$ norm: $\|p_0\|_{L^2(\Omega_0)} = \inf_{q\in S_0}\|q\|_{L^2(\Omega_0)}$.*

*Proof.* The closedness and convexity of $S_0$ are easy to deduce. Here we only prove (18). Take $q = p_0$ in (8), $q = p_\varepsilon$ in (17) for $p = p_0$, and add the two inequalities to obtain

$$\varepsilon(p_\varepsilon, p_0 - p_\varepsilon)_{L^2(\Omega_0)} \geq \sum_{i=1}^{i_0} w_i \|u_i(p_\varepsilon - p_0)\|^2_{L^2(\Gamma_0)}. \tag{19}$$

Thus, $(p_\varepsilon, p_0 - p_\varepsilon)_{L^2(\Omega_0)} \geq 0$, $\|p_\varepsilon\|_{L^2(\Omega_0)} \leq \|p_0\|_{L^2(\Omega_0)}$, and $\{p_\varepsilon\}$ is uniformly bounded. Let $\{p_{\varepsilon'}\}$ be a subsequence of $\{p_\varepsilon\}$, converging weakly to $p$. Since $S_0$ is weakly closed, $p \in S_0$. Moreover, $\|p\|_{L^2(\Omega_0)} \leq \liminf_{\varepsilon'\to 0}\|p_{\varepsilon'}\|_{L^2(\Omega_0)} \leq \|p_0\|_{L^2(\Omega_0)}$. Now $p_0$ is the unique element in $S_0$ with minimal $L^2(\Omega_0)$ norm, $p = p_0$. Thus the limit $p = p_0$ does not depend on the subsequence selected; consequently, $p_\varepsilon$ converges weakly to $p_0$ in $L^2(\Omega_0)$ as $\varepsilon \to 0$. Using the relation

$$\begin{aligned} \|p_\varepsilon - p_0\|^2_{L^2(\Omega_0)} &= \|p_\varepsilon\|^2_{L^2(\Omega_0)} - 2(p_\varepsilon, p_0)_{L^2(\Omega_0)} + \|p_0\|^2_{L^2(\Omega_0)} \\ &\leq 2\|p_0\|^2_{L^2(\Omega_0)} - 2(p_\varepsilon, p_0)_{L^2(\Omega_0)}, \end{aligned} \tag{20}$$

we conclude the strong convergence $\|p_\varepsilon - p_0\|_{L^2(\Omega_0)} \to 0$ as $\varepsilon \to 0$. □

As a simple consequence of Proposition 1, if the solution set $S_0 = \{p\}$ is a singleton, then $p_\varepsilon \to p$ in $L^2(\Omega)$, as $\varepsilon \to 0$. Note that if $Q_{\text{ad}}$ is a bounded set, then $S_0$ is nonempty. This follows from applying a standard result on convex minimization, for example, [17, Theorem 3.3.12]. As in the first part of the proof of Theorem 1, $L^2(\Omega_0)$ is a Hilbert space, $Q_{\text{ad}} \subset L^2(\Omega_0)$ is convex and closed, $J_\varepsilon|_{\varepsilon=0} : Q_{\text{ad}} \to \mathbb{R}$ is convex and continuous. Since $Q_{\text{ad}}$ is assumed to be bounded, Problem 1 with $\varepsilon = 0$ has a solution. Without further information on $Q_{\text{ad}}$, though, we cannot ascertain uniqueness of a solution when $\varepsilon = 0$.

### 3. NUMERICAL APPROXIMATIONS

In this section, we discretize Problem 1 and derive error estimates for the numerical solutions. First we need to discretize the boundary value problem (5). Let $\{\mathcal{T}_h\}$ ($h$: meshsize) be a regular family of finite element partitions of $\overline{\Omega}$ into triangular/tetrahedral elements such that each element at the boundary $\Gamma$ has at most one nonstraight face (for a three-dimensional domain) or side (for a two-dimensional domain). For each triangulation $\mathcal{T}_h = \{K\}$, let $V^h \subset V$ be the linear element space. For any $q \in L^2(\Omega_0)$, denote by $u_i^h = u_i^h(q) \in V^h$ the solution of the problem

$$\begin{aligned} \int_\Omega \big(D_i\nabla u_i^h \cdot \nabla v^h + \mu_{a,i}u_i^h v^h\big)dx + \int_\Gamma \frac{1}{2A}u_i^h v^h ds \\ = \int_{\Omega_0} \omega_i q v^h dx \quad \forall v^h \in V^h. \end{aligned} \tag{21}$$

The solution $u_i^h(q)$ exists and is unique. Let

$$J_\varepsilon^h(q) = \sum_{i=1}^{i_0} w_i \|u_i^h(q) - 2AQ_i\|^2_{L^2(\Gamma_0)} + \varepsilon\|q\|^2_{L^2(\Omega_0)}. \tag{22}$$

The admissible source function space $Q_{\text{ad}}$ may or may not need to be discretized. In general, let $Q_{\text{ad},1} \subset Q_{\text{ad}}$ be nonempty, closed and convex. Later in the section, we will consider two possible choices of $Q_{\text{ad},1}$. We then introduce the following discretization of Problem 1.

*Problem 2.* Find $p_\varepsilon^h \in Q_{\text{ad},1}$ such that $J_\varepsilon^h(p_\varepsilon^h) = \inf_{q\in Q_{\text{ad},1}} J_\varepsilon^h(q)$.

Similar to Theorem 1 and Proposition 1, we have the following result.

**Proposition 2.** *For $\varepsilon > 0$, Problem 2 has a unique solution $p_\varepsilon^h \in Q_{\text{ad},1}$, which is characterized by the discrete variational inequality:*

$$\begin{aligned} \sum_{i=1}^{i_0} w_i\big(u_i^h(p_\varepsilon^h) - 2AQ_i, u_i^h(q - p_\varepsilon^h)\big)_{L^2(\Gamma_0)} \\ + \varepsilon(p_\varepsilon^h, q - p_\varepsilon^h)_{L^2(\Omega_0)} \geq 0 \quad \forall q \in Q_{\text{ad},1}. \end{aligned} \tag{23}$$

*If $Q_{\text{ad},1}$ is a subspace of $L^2(\Omega_0)$, then $p_\varepsilon^h$ is characterized by a variational equation:*

$$\sum_{i=1}^{i_0} w_i\big(u_i^h(p_\varepsilon^h) - 2AQ_i, u_i^h(q)\big)_{L^2(\Gamma_0)} + \varepsilon(p_\varepsilon^h, q)_{L^2(\Omega_0)} = 0 \quad \forall q \in Q_{\text{ad},1}. \tag{24}$$

*The solution $p_\varepsilon^h$ depends continuously on the data.*

*Assume the solution set $S_0^h \neq \emptyset$ for Problem 2 with $\varepsilon = 0$. Then $S_0^h \subset Q_{\text{ad},1}$ is closed and convex, and $p_\varepsilon^h \to p_0^h$ in $L^2(\Omega_0)$ as $\varepsilon \to 0$, where $p_0^h \in S_0^h$ satisfies $\|p_0^h\|_{L^2(\Omega_0)} = \inf_{q\in S_0^h}\|q\|_{L^2(\Omega_0)}$.*

If $Q_{\text{ad},1}$ is a bounded set, then $S_0^h$ is nonempty. In concrete situations, it is possible to show the nonemptyness of the solution set $S_0^h$ directly.

We then turn to error estimation. For this purpose, we further assume

$$\begin{aligned} \Gamma \in C^{1,1}, \qquad A \in C^{0,1}(\Gamma), \\ D_i \in C^{0,1}(\Omega), \quad \mu_{a,i} \in L^\infty(\Omega), \quad 1 \leq i \leq i_0. \end{aligned} \tag{25}$$

Then we have the solution regularity bound ([22, Theorems 2.3.3.6 & 2.4.2.6])

$$\|u_i(q)\|_{H^2(\Omega)} \le c\|q\|_{L^2(\Omega_0)}. \tag{26}$$

The assumptions (25) are made to ensure the validity of the solution regularity (26) used below in error estimation. Without the solution regularity property, error estimates with lower convergence orders can still be derived. Let $\Pi_{V^h} u \in V^h$ be the piecewise linear interpolant of $u$. We have the finite element interpolation error estimate:

$$\|u - \Pi_{V^h} u\|_{L^2(\Omega)} + h\|u - \Pi_{V^h} u\|_{H^1(\Omega)} \le \mathrm{ch}^2 \|u\|_{H^2(\Omega)} \quad \forall u \in H^2(\Omega). \tag{27}$$

This error estimate is usually proved when $\Omega$ is a polyhedral/polygonal domain so that each element $K$ in a finite element partition $\mathcal{T}_h$ has straight faces/sides on its boundary (e.g., [23, 24]). For applications in bioluminescence tomography, $\Omega$ is a smooth domain, and is not polyhedral. In such an application, the error estimate (27) still holds [8]. For the finite element solution of (21), there is a constant $c > 0$ independent of $h$ and $\varepsilon$ such that

$$\|u_i(q) - u_i^h(q)\|_{L^2(\Gamma)} \le \mathrm{ch}^{3/2} \|q\|_{L^2(\Omega_0)} \quad \forall q \in L^2(\Omega_0). \tag{28}$$

This error bound is shown in [8] in a somewhat different setting.

Now we distinguish two cases in the discretization of the admissible set $Q_{\mathrm{ad},1}$. We first consider the case $Q_{\mathrm{ad},1} = Q_{\mathrm{ad}}$. This is the natural choice when $Q_{\mathrm{ad}}$ is a finite dimensional subspace or subset of linear combinations of specified functions such as the characteristic functions of certain subsets of $\Omega$. We have the following error bound.

**Theorem 3.** *With $Q_{\mathrm{ad},1} = Q_{\mathrm{ad}}$, there is a constant $c > 0$ independent of $\varepsilon$ and $h$ such that*

$$\begin{aligned}\max_{1\le i\le i_0} &\|u_i(p_\varepsilon) - u_i^h(p_\varepsilon^h)\|_{L^2(\Gamma_0)} + \varepsilon^{1/2}\|p_\varepsilon - p_\varepsilon^h\|_{L^2(\Omega_0)} \\ &\le \mathrm{ch}^{3/4} \sum_{i=1}^{i_0} \|u_i(p_\varepsilon) - 2AQ_i\|_{L^2(\Gamma_0)}^{1/2} \|p_\varepsilon - p_\varepsilon^h\|_{L^2(\Omega_0)}^{1/2} \\ &\quad + \mathrm{ch}^{3/2} \|p_\varepsilon\|_{L^2(\Omega_0)}.\end{aligned} \tag{29}$$

*Proof.* We choose $q = p_\varepsilon$ in (23), $q = p_\varepsilon^h$ in (8), and use the two inequalities to obtain

$$\begin{aligned}\sum_{i=1}^{i_0} &w_i\|u_i(p_\varepsilon) - u_i^h(p_\varepsilon^h)\|_{L^2(\Gamma_0)}^2 + \varepsilon\|p_\varepsilon - p_\varepsilon^h\|_{L^2(\Omega_0)}^2 \\ &\le \sum_{i=1}^{i_0} w_i\big(u_i(p_\varepsilon) - u_i^h(p_\varepsilon^h), u_i(p_\varepsilon) - u_i^h(p_\varepsilon)\big)_{L^2(\Gamma_0)} \\ &\quad + \sum_{i=1}^{i_0} w_i\big(u_i(p_\varepsilon) - 2AQ_i, u_i^h(p_\varepsilon - p_\varepsilon^h) - u_i(p_\varepsilon - p_\varepsilon^h)\big)_{L^2(\Gamma_0)}.\end{aligned} \tag{30}$$

Then,

$$\begin{aligned}\sum_{i=1}^{i_0} &w_i\|u_i(p_\varepsilon) - u_i^h(p_\varepsilon^h)\|_{L^2(\Gamma_0)}^2 + \varepsilon\|p_\varepsilon - p_\varepsilon^h\|_{L^2(\Omega_0)}^2 \\ &\le c\sum_{i=1}^{i_0} w_i\|u_i(p_\varepsilon) - u_i^h(p_\varepsilon)\|_{L^2(\Gamma_0)}^2 \\ &\quad + c\sum_{i=1}^{i_0} w_i\|u_i(p_\varepsilon) - 2AQ_i\|_{L^2(\Gamma_0)} \\ &\quad \times\|u_i(p_\varepsilon - p_\varepsilon^h) - u_i^h(p_\varepsilon - p_\varepsilon^h)\|_{L^2(\Gamma_0)}.\end{aligned} \tag{31}$$

The error bound (29) follows from this inequality together with (28). □

Further error bounds require more information on the data. We present two sample results as consequences of Theorem 3.

Assume $Q_{\mathrm{ad}}$ is a bounded set in $L^2(\Omega)$. Then $\|p_\varepsilon\|_{L^2(\Omega_0)}$ and $\|p_\varepsilon^h\|_{L^2(\Omega_0)}$ are uniformly bounded with respect to $\varepsilon$ and $h$. By (26), $\|u_i(p_\varepsilon)\|_{H^2(\Omega)}$, and hence $\|u_i(p_\varepsilon)\|_{L^2(\Gamma)}$ as well, is uniformly bounded. So from (29), we see that there is a constant $c > 0$ independent of $\varepsilon$ and $h$ such that

$$\max_{1\le i\le i_0} \|u_i(p_\varepsilon) - u_i^h(p_\varepsilon^h)\|_{L^2(\Gamma_0)} + \varepsilon^{1/2}\|p_\varepsilon - p_\varepsilon^h\|_{L^2(\Omega_0)} \le \mathrm{ch}^{3/4}. \tag{32}$$

Next, we assume the data are compatible in the sense that there exists $p_1 \in Q_{\mathrm{ad}}$ such that $u_i(p_1) = 2AQ_i$ on $\Gamma_0$ for $1 \le i \le i_0$. Under this assumption, we have

$$\begin{aligned}\sum_{i=1}^{i_0} w_i\|u_i(p_\varepsilon) - 2AQ_i\|_{L^2(\Gamma_0)}^2 + \varepsilon\|p_\varepsilon\|_{L^2(\Omega_0)}^2 &\le J_\varepsilon(p_1) \\ &= \varepsilon\|p_1\|_{L^2(\Omega_0)}^2,\end{aligned} \tag{33}$$

and so for all $\varepsilon > 0$, $\|p_\varepsilon\|_{L^2(\Omega_0)} + \varepsilon^{-1/2}\|u_i(p_\varepsilon) - 2AQ_i\|_{L^2(\Gamma_0)} \le 2\|p_1\|_{L^2(\Omega_0)}$. Thus from (29),

$$\begin{aligned}\max_{1\le i\le i_0} &\|u_i(p_\varepsilon) - u_i^h(p_\varepsilon^h)\|_{L^2(\Gamma_0)} + \varepsilon^{1/2}\|p_\varepsilon - p_\varepsilon^h\|_{L^2(\Omega_0)} \\ &\le \mathrm{ch}^{3/4}\varepsilon^{1/4}\|p_\varepsilon - p_\varepsilon^h\|_{L^2(\Omega_0)}^{1/2} + \mathrm{ch}^{3/2}.\end{aligned} \tag{34}$$

The first term on the right-hand side is bounded as follows:

$$\mathrm{ch}^{3/4}\varepsilon^{1/4}\|p_\varepsilon - p_\varepsilon^h\|_{L^2(\Omega_0)}^{1/2} \le \frac{1}{2}\varepsilon^{1/2}\|p_\varepsilon - p_\varepsilon^h\|_{L^2(\Omega_0)} + \mathrm{ch}^{3/2}. \tag{35}$$

Therefore, we conclude that for some constant $c > 0$ independent of $\varepsilon$ and $h$,

$$\max_{1\le i\le i_0} \|u_i(p_\varepsilon) - u_i^h(p_\varepsilon^h)\|_{L^2(\Gamma_0)} + \varepsilon^{1/2}\|p_\varepsilon - p_\varepsilon^h\|_{L^2(\Omega_0)} \le \mathrm{ch}^{3/2}. \tag{36}$$

We now consider the situation where $Q_{\text{ad}}$ is a general admissible set and needs to be discretized. In addition to the regular family of finite element partitions $\{\mathcal{T}_h\}$ of $\overline{\Omega}$, let $\{\mathcal{T}_{0,H}\}$ be a regular family of finite element partitions of $\overline{\Omega_0}$ such that each element at the boundary $\partial\Omega_0$ has at most one nonstraight face (for a three-dimensional domain) or side (for a two-dimensional domain). The partitions $\mathcal{T}_h$ and $\mathcal{T}_{0,H}$ do not need to be related; however, $\mathcal{T}_h$ can be constructed based on $\mathcal{T}_{0,H}$. Let $Q^H \subset L^2(\Omega_0)$ be the piecewise constant space. Define $Q_{\text{ad},1} = Q^H_{\text{ad}} \equiv Q^H \cap Q_{\text{ad}}$. We denote the solution of Problem 2 by $p^{h,H}_\varepsilon$.

Denote by $\Pi^H : L^2(\Omega_0) \to Q^H$ the orthogonal projection opeartor: for $q \in L^2(\Omega_0)$,

$$\Pi^H q \in Q^H, \quad (\Pi^H q, q^H)_{L^2(\Omega_0)} = (q, q^H)_{L^2(\Omega_0)} \quad \forall q^H \in Q^H. \tag{37}$$

We will use the following properties:

$$||\Pi^H q||_{L^2(\Omega_0)} \le ||q||_{L^2(\Omega_0)} \quad \forall q \in L^2(\Omega_0), \tag{38}$$

$$||q - \Pi^H q||_{L^2(\Omega_0)} \le cH|q|_{H^1(\Omega_0)} \quad \forall q \in H^1(\Omega_0), \tag{39}$$

$$\begin{aligned}\int_{\Omega_0} (q - \Pi^H q) v\, dx \\ \le cH||q - \Pi^H q||_{L^2(\Omega_0)} ||v||_{H^1(\Omega)} \quad \forall v \in H^1(\Omega).\end{aligned} \tag{40}$$

By the elementwise formula

$$\Pi^H q|_K = \frac{1}{|K|}\int_K q\, dx \quad \forall K \in \mathcal{T}_{0,H}, \tag{41}$$

since $Q_{\text{ad}} \subset L^2(\Omega_0)$ is convex, we see that $\Pi^H : Q_{\text{ad}} \to Q^H_{\text{ad}}$, that is, for $q \in Q_{\text{ad}}$, its piecewise constant orthogonal projection $\Pi^H q \in Q^H_{\text{ad}}$. We first present a preparatory result.

**Lemma 1.** *There is a constant $c > 0$ independent of $h$ and $H$ such that*

$$||u_i(q) - u_i^h(\Pi^H q)||_{H^1(\Omega)} \le cH||q - \Pi^H q||_{L^2(\Omega_0)} + \text{ch}\, ||q||_{L^2(\Omega_0)}. \tag{42}$$

*Proof.* From the definitions of $u_i(q)$ and $u_i^h(\Pi^H q)$, we have

$$\begin{aligned}&\int_\Omega [D_i \nabla(u_i(q) - u_i^h(\Pi^H q)) \cdot \nabla v^h \\ &\quad + \mu_{a,i}(u_i(q) - u_i^h(\Pi^H q)) v^h] dx \\ &\quad + \int_\Gamma \frac{1}{2A}[u_i(q) - u_i^h(\Pi^H q)] v^h ds \\ &= \int_{\Omega_0} \omega_i (q - \Pi^H q) v^h\, dx \quad \forall v^h \in V^h.\end{aligned} \tag{43}$$

For any $v^h \in V^h$, write

$$\begin{aligned}&\int_\Omega [D_i |\nabla(u_i(q) - u_i^h(\Pi^H q))|^2 + \mu_{a,i} |u_i(q) - u_i^h(\Pi^H q)|^2] dx \\ &\quad + \int_\Gamma \frac{1}{2A} |u_i(q) - u_i^h(\Pi^H q)|^2 ds \\ &= \int_\Omega [D_i \nabla(u_i(q) - u_i^h(\Pi^H q)) \cdot \nabla(u_i(q) - v^h) \\ &\quad + \mu_{a,i}(u_i(q) - u_i^h(\Pi^H q))(u_i(q) - v^h)] dx \\ &\quad + \int_\Gamma \frac{1}{2A}(u_i(q) - u_i^h(\Pi^H q))(u_i(q) - v^h) ds \\ &\quad + \int_\Gamma \frac{1}{2A}(u_i(q) - u_i^h(\Pi^H q))(v^h - u_i^h(\Pi^H q)) ds \\ &\quad + \int_\Omega [D_i \nabla(u_i(q) - u_i^h(\Pi^H q)) \cdot \nabla(v^h - u_i^h(\Pi^H q)) \\ &\quad + \mu_{a,i}(u_i(q) - u_i^h(\Pi^H q))(v^h - u_i^h(\Pi^H q))] dx.\end{aligned} \tag{44}$$

The sum of the last two integrals on the right-hand side can be replaced by the following, with the help of (43), $\int_{\Omega_0} \omega_i (q - \Pi^H q)(v^h - u_i(q) + u_i(q) - u_i^h(\Pi^H q))\, dx$, which is bounded by (40). Then after some algebraic manipulations we obtain

$$\begin{aligned}&||u_i(q) - u_i^h(\Pi^H q)||_{H^1(\Omega)} \\ &\quad \le c\left[\inf_{v^h \in V^h} ||u_i(q) - v^h||_{H^1(\Omega)} + H||q - \Pi^H q||_{L^2(\Omega_0)}\right].\end{aligned} \tag{45}$$

We use the error bound $\inf_{v^h \in V^h} ||u_i(q) - v^h||_{H^1(\Omega)} \le \text{ch}\, |u_i(q)|_{H^2(\Omega)}$ and the regularity bound (26) in (45) to obtain (42). □

We now prove the following error estimate.

**Theorem 4.** *With the choice $Q_{\text{ad},1} = Q^H_{\text{ad}}$ as a subset of piecewise constant functions, there is a constant $c > 0$ independent of $\varepsilon$, $h$, and $H$ such that*

$$\begin{aligned}&\max_{1 \le i \le i_0} ||u_i(p_\varepsilon) - u_i^h(p^{h,H}_\varepsilon)||_{L^2(\Gamma_0)} + \varepsilon^{1/2}||p_\varepsilon - p^{h,H}_\varepsilon||_{L^2(\Omega_0)} \\ &\quad \le c\sum_{i=1}^{i_0} ||u_i(p_\varepsilon) - 2AQ_i||^{1/2}_{L^2(\Gamma_0)} \\ &\qquad \times (H^{1/2}||p_\varepsilon - \Pi^H p_\varepsilon||^{1/2}_{L^2(\Omega_0)} + h^{1/2}||p_\varepsilon||^{1/2}_{L^2(\Omega_0)} \\ &\qquad + h^{3/4}||p^{h,H}_\varepsilon||^{1/2}_{L^2(\Omega_0)}) \\ &\qquad + cH||p_\varepsilon - \Pi^H p_\varepsilon||_{L^2(\Omega_0)} + \text{ch}\, ||p_\varepsilon||_{L^2(\Omega_0)}.\end{aligned} \tag{46}$$

*Proof.* Similar to the first part of the proof of Theorem 3, there holds

$$\begin{aligned}

&\sum_{i=1}^{i_0} w_i \|u_i(p_\varepsilon) - u_i^h(p_\varepsilon^{h,H})\|^2_{L^2(\Gamma_0)} + \varepsilon\|p_\varepsilon - p_\varepsilon^{h,H}\|^2_{L^2(\Omega_0)} \\

&\quad \le \sum_{i=1}^{i_0} w_i \left(u_i(p_\varepsilon) - u_i^h(p_\varepsilon^{h,H}), u_i(p_\varepsilon) - u_i^h(\Pi^H p_\varepsilon)\right)_{L^2(\Gamma_0)} \\

&\qquad + \sum_{i=1}^{i_0} w_i \big(u_i(p_\varepsilon) - 2AQ_i, u_i^h(\Pi^H p_\varepsilon) \\

&\qquad\qquad - u_i(p_\varepsilon) + u_i(p_\varepsilon^{h,H}) - u_i^h(p_\varepsilon^{h,H})\big)_{L^2(\Gamma_0)},

\end{aligned} \tag{47}$$

where we used the property $(p_\varepsilon^{h,H}, \Pi^H p_\varepsilon - p_\varepsilon)_{L^2(\Omega_0)} = 0$. Then,

$$\begin{aligned}

&\sum_{i=1}^{i_0} w_i \|u_i(p_\varepsilon) - u_i^h(p_\varepsilon^{h,H})\|^2_{L^2(\Gamma_0)} + \varepsilon\|p_\varepsilon - p_\varepsilon^{h,H}\|^2_{L^2(\Omega_0)} \\

&\quad \le c\sum_{i=1}^{i_0} \|u_i(p_\varepsilon) - u_i^h(\Pi^H p_\varepsilon)\|^2_{L^2(\Gamma_0)} \\

&\qquad + c\sum_{i=1}^{i_0} \|u_i(p_\varepsilon) - 2AQ_i\|_{L^2(\Gamma_0)} \\

&\qquad \times \big(\|u_i(p_\varepsilon) - u_i^h(\Pi^H p_\varepsilon)\|_{L^2(\Gamma_0)} \\

&\qquad\quad + \|u_i(p_\varepsilon^{h,H}) - u_i^h(p_\varepsilon^{h,H})\|_{L^2(\Gamma_0)}\big).

\end{aligned} \tag{48}$$

Applying the error bound (28) and Lemma 1, we can then deduce (46). □

Similar to Theorem 3, we present two sample results as consequences of Theorem 4. If $Q_{\text{ad}}$ is bounded in $L^2(\Omega_0)$, then there is a constant $c > 0$ independent of $\varepsilon$, $h$, and $H$ such that

$$\begin{aligned}

&\max_{1\le i\le i_0} \|u_i(p_\varepsilon) - u_i^h(p_\varepsilon^{h,H})\|_{L^2(\Gamma_0)} + \varepsilon^{1/2}\|p_\varepsilon - p_\varepsilon^{h,H}\|_{L^2(\Omega_0)} \\

&\qquad \le c\left(H^{1/2}\|p_\varepsilon - \Pi^H p_\varepsilon\|^{1/2}_{L^2(\Omega_0)} + h^{3/4}\right).

\end{aligned} \tag{49}$$

If the data are compatible, then there is a constant $c > 0$ independent of $\varepsilon$, $h$, and $H$ such that

$$\begin{aligned}

&\max_{1\le i\le i_0} \|u_i(p_\varepsilon) - u_i^h(p_\varepsilon^{h,H})\|_{L^2(\Gamma_0)} + \varepsilon^{1/2}\|p_\varepsilon - p_\varepsilon^{h,H}\|_{L^2(\Omega_0)} \\

&\qquad \le c\left(h + h^{1/2}\varepsilon^{1/4} + H\|p_\varepsilon - \Pi^H p_\varepsilon\|_{L^2(\Omega_0)}\right).

\end{aligned} \tag{50}$$

These error bounds involve an approximation error term $\|p_\varepsilon - \Pi^H p_\varepsilon\|_{L^2(\Omega_0)}$. This term can always be bounded by $\|p_\varepsilon\|_{L^2(\Omega_0)} + \|\Pi^H p_\varepsilon\|_{L^2(\Omega_0)} \le 2\|p_\varepsilon\|_{L^2(\Omega_0)} \le c$. Moreover, as is shown in [8], if $S_0 \ne \emptyset$, then $\|p_\varepsilon - \Pi^H p_\varepsilon\|_{L^2(\Omega_0)} \to 0$ as $H, \varepsilon \to 0$, and if $p_\varepsilon \in H^1(\Omega_0)$, then $\|p_\varepsilon - \Pi^H p_\varepsilon\|_{L^2(\Omega_0)} \le cH\|p_\varepsilon\|_{H^1(\Omega_0)}$.

We underline that the above theoretical results on the numerical solutions with the second choice of $Q_{\text{ad},1}$ are still valid if $Q^H \subset L^2(\Omega_0)$ is a general finite element space containing piecewise constants. The proofs of the results are the same as long as we define $\Pi^H$ to be the orthogonal projection operator in $L^2(\Omega_0)$ onto the space of piecewise constants.

When the regularization parameter $\varepsilon$ is chosen related to the discretization parameters $h$ and $H$, we may express the error bounds in terms of the discretization parameters only. For example, from (36), $\max_{1\le i\le i_0} \|u_i(p_\varepsilon) - u_i^h(p_\varepsilon^h)\|_{L^2(\Gamma_0)} \le \text{ch}^{3/2}$, and if $\varepsilon = \text{ch}^\beta$, $0 < \beta < 3$ in (36), then $|p_\varepsilon - p_\varepsilon^h\|_{L^2(\Omega_0)} \le \text{ch}^{(3-\beta)/2}$.

Finally, we comment that when the solution set $S_0$ is nonempty, convergence of the numerical solution $p_\varepsilon^h$ to the minimal energy solution $p_0 \in S_0$ follows from the triangle inequality

$$\|p_\varepsilon^h - p_0\|_{L^2(\Omega_0)} \le \|p_\varepsilon - p_0\|_{L^2(\Omega_0)} + \|p_\varepsilon - p_\varepsilon^h\|_{L^2(\Omega_0)} \tag{51}$$

together with (18) and the convergence of $p_\varepsilon^h$ to $p_\varepsilon$ in $L^2(\Omega_0)$. A similar statement holds for the convergence of $p_\varepsilon^{h,H}$ to $p_0 \in S_0$.

### 4. NUMERICAL SIMULATION

For a numerical simulation of source reconstruction, we consider a cylindrical phantom with a diameter 20 mm and a height 20 mm. We choose the coordinate system so that the phantom is represented as

$$\Omega = \{\mathbf{x} = (x_1, x_2, x_3)^T \in \mathbb{R}^3 \mid x_1^2 + x_2^2 < 100,\ 0 < x_3 < 20\}. \tag{52}$$

The measurement boundary is the entire lateral side of the cylinder:

$$\Gamma_0 = \{\mathbf{x} = (x_1, x_2, x_3)^T \in \mathbb{R}^3 \mid x_1^2 + x_2^2 = 100,\ 0 \le x_3 \le 20\}. \tag{53}$$

Based on the results of the bioluminescent spectral analysis experiment in [15], we split the spectrum into three regions: $\Lambda_1 = [400\,\text{nm}, 530\,\text{nm})$, $\Lambda_2 = [530\,\text{nm}, 630\,\text{nm})$, $\Lambda_3 = [630\,\text{nm}, 750\,\text{nm}]$, and quantify the energy distribution weights to be $\omega_1 = 0.29$ in $\Lambda_1$, $\omega_2 = 0.48$ in $\Lambda_2$, and $\omega_3 = 0.23$ in $\Lambda_3$. The optical parameters are assigned as follows (unit: $\text{mm}^{-1}$):

$$\mu_a = \begin{cases} 0.014 & \text{in } \Lambda_1, \\ 0.0104 & \text{in } \Lambda_2, \\ 0.0075 & \text{in } \Lambda_3; \end{cases} \qquad \mu_s' = \begin{cases} 0.75 & \text{in } \Lambda_1, \\ 0.85 & \text{in } \Lambda_2, \\ 1.05 & \text{in } \Lambda_3. \end{cases} \tag{54}$$

The refractive index $A$ is 1.37 in the biological tissue.

The phantom is discretized into 58161 tetrahedral elements and 10778 nodes. A total of 2170 datum nodes are distributed along $\Gamma_0$, and simulated measurement data of photon density at datum nodes are generated from the diffusion approximation model. Two uniform light sources are embedded into the phantom. The first light source has a power 2.2 nano-Watts and is distributed in those elements whose

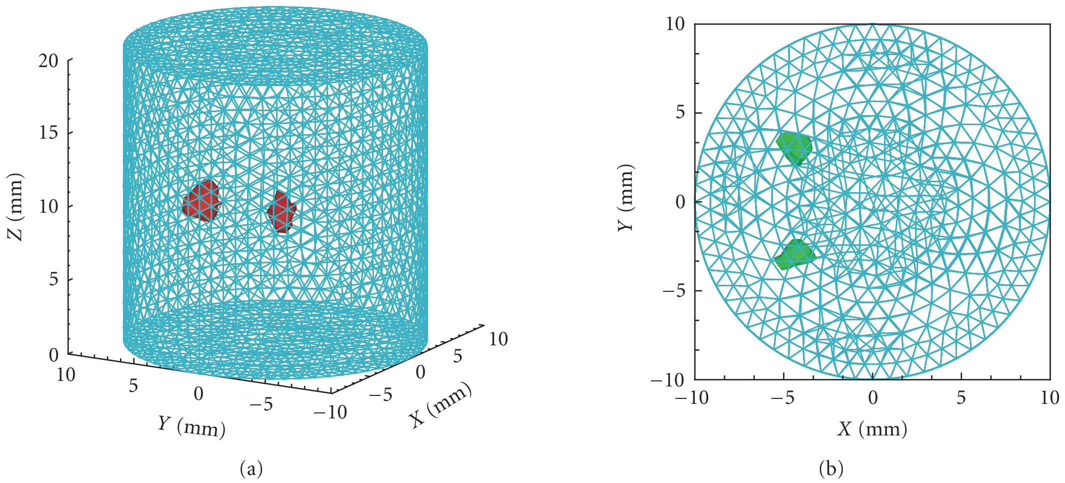

FIGURE 1: True light source distribution: (a) 3D view, (b) 2D view.

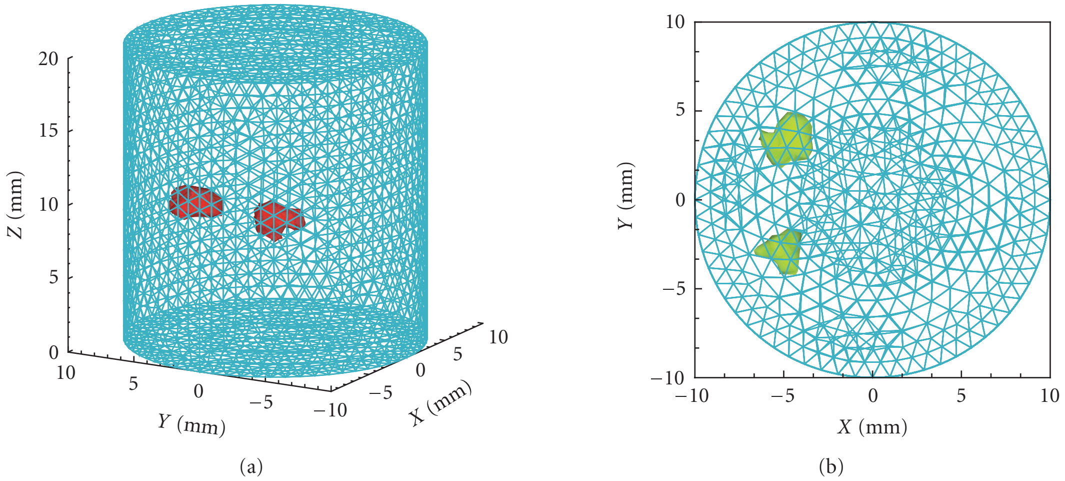

FIGURE 2: Reconstructed light source distribution with a 5% random noise in surface measurement data: (a) 3D view, (b) 2D view.

vertices have a distance less than or equal to 2 mm from $(-4,-3,10)^T$. The second light source has a power 2.4 nano-Watts and is distributed in those elements whose vertices have a distance less than or equal to 2 mm from $(-4,3,10)^T$. Denote the region where the true light sources are distributed to be $D_{\text{tr}}$. Figure 1 shows the finite element mesh, the 3D and 2D views of the true source distribution in the phantom. Here and in the following figures, the 2D view is for the midsection ($x_3 = 10$) of the cylinder.

We choose the permissible region

$$\Omega_0 = \{\mathbf{x} \in \Omega \mid x_1 < 0,\ -6 < x_2 < 6,\ 9 < x_3 < 12\}, \quad (55)$$

and use piecewise constants to approximate the source density function. In simulating the difference between discretized solution $u^h$ and solution $u$ of the boundary value problem (1)-(2), we introduce random noises of the sizes 5%, 10%, and 20% in the datasets on the lateral surface covering phantom. Then the regularization method with $\varepsilon = 1.0 \times 10^{-8}$ is applied to reconstruct source distribution. A modified Newton method and an active set strategy with simple constraint are used in solving the discrete Problem 2. The reconstructed results show that with random noises of sizes 5%, 10%, and 20%, we have 80%, 77%, and 76% of the total power of the reconstructed light sources located in $D_{\text{tr}}$, the region of the true light source. Figures 2–4 illustrate 3D and 2D views of the reconstructed light source distribution with 5%, 10%, and 20% random noises in surface flux density, respectively. The numerical results show that the numerical method is computationally efficient, stable, and robust with respect to noise in the measured data.

### 5. CONCLUDING REMARK

Multispectral bioluminescence tomography is a new development in optical imaging and has a great potential

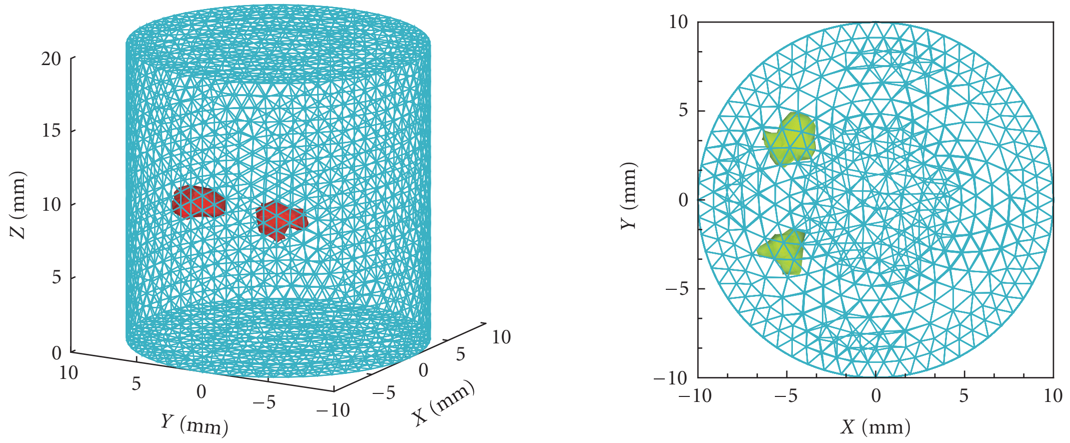

(a) (b)

FIGURE 3: Reconstructed light source distribution with a 10% random noise in surface measurement data: (a) 3D view, (b) 2D view.

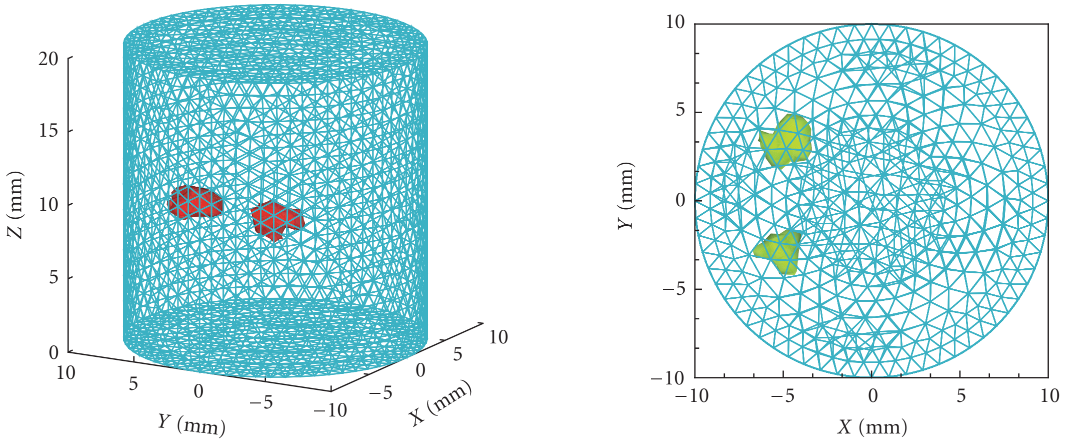

(a) (b)

FIGURE 4: Reconstructed light source distribution with a 20% random noise in surface measurement data: (a) 3D view, (b) 2D view.

to advance the field of biomedical imaging. In this paper, we have established a mathematical framework for studies of multispectral bioluminescence tomography. We have analyzed the theoretical properties of the multispectral imaging model including the solution existence, uniqueness, and continuous dependence on the data. We have rigorously demonstrated the convergence and error bounds for the discrete source functions that are obtained by minimizing the discretized objective functions subject to the PDE constraints. Numerical examples have illustrated the performance of the numerical scheme for multispectral bioluminescence tomography.

### ACKNOWLEDGMENTS

This work was supported by an NIH Grant EB001685. We thank the referees for their valuable comments.

### REFERENCES

[1] B. W. Rice, M. D. Cable, and M. B. Nelson, "In vivo imaging of light-emitting probes," *Journal of Biomedical Optics*, vol. 6, no. 4, pp. 432–440, 2001.

[2] C. H. Contag and M. H. Bachmann, "Advances in in vivo bioluminescence imaging of gene expression," *Annual Review of Biomedical Engineering*, vol. 4, pp. 235–260, 2002.

[3] G. Wang, E. A. Hoffman, G. McLennan, et al., "Development of the first bioluminescent CT scanner," *Radiology*, vol. 229(P), p. 566, 2003.

[4] G. Wang, Y. Li, and M. Jiang, "Uniqueness theorems in bioluminescence tomography," *Medical Physics*, vol. 31, no. 8, pp. 2289–2299, 2004.

[5] M. Jiang and G. Wang, "Image reconstruction for bioluminescence tomography," in *Developments in X-Ray Tomography IV*, vol. 5535 of *Proceedings of SPIE*, pp. 335–351, 2004.

[6] W. Cong, G. Wang, D. Kumar, et al., "Practical reconstruction method for bioluminescence tomography," *Optics Express*, vol. 13, no. 18, pp. 6756–6771, 2005.

[7] A. Cong and G. Wang, "Multispectral bioluminescence tomography: methodology and simulation," *International Journal of Biomedical Imaging*, vol. 2006, Article ID 57614, 7 pages, 2006.
[8] W. Han, W. Cong, and G. Wang, "Mathematical theory and numerical analysis of bioluminescence tomography," *Inverse Problems*, vol. 22, pp. 1659–1675, 2006.
[9] X. Gu, Q. Zhang, L. Larcom, and H. Jiang, "Three-dimensional bioluminescence tomography with model-based reconstruction," *Optics Express*, vol. 12, no. 17, pp. 3996–4000, 2004.
[10] C. Kuo, O. Coquoz, T. Troy, D. Zwarg, and B. Rice, "Bioluminescent tomography for in vivo localization and quantification of luminescent sources from a multiple-view imaging system," *Molecular Imaging*, vol. 4, p. 370, 2005.
[11] G. Alexandrakis, F. R. Rannou, and A. F. Chatziioannou, "Tomographic bioluminescence imaging by use of a combined optical-PET (OPET) system: a computer simulation feasibility study," *Physics in Medicine and Biology*, vol. 50, no. 17, pp. 4225–4241, 2005.
[12] A. J. Chaudhari, F. Darvas, J. R. Bading, et al., "Hyperspectral and multispectral bioluminescence optical tomography for small animal imaging," *Physics in Medicine and Biology*, vol. 50, no. 23, pp. 5421–5441, 2005.
[13] N. V. Slavine, M. A. Lewis, E. Richer, and P. P. Antich, "Iterative reconstruction method for light emitting sources based on the diffusion equation," *Medical Physics*, vol. 33, no. 1, pp. 61–68, 2006.
[14] H. Dehghani, S. C. Davis, S. Jiang, B. W. Pogue, K. D. Paulsen, and M. S. Patterson, "Spectrally resolved bioluminescence optical tomography," *Optics Letters*, vol. 31, no. 3, pp. 365–367, 2006.
[15] H. Zhao, T. C. Doyle, O. Coquoz, F. Kalish, B. W. Rice, and C. H. Contag, "Emission spectra of bioluminescent reporters and interaction with mammalian tissue determine the sensitivity of detection in vivo," *Journal of Biomedical Optics*, vol. 10, no. 4, Article ID 041210, 9 pages, 2005.
[16] M. Schweiger, S. R. Arridge, M. Hiraoka, and D. T. Delpy, "The finite element method for the propagation of light in scattering media: boundary and source conditions," *Medical Physics*, vol. 22, no. 11 I, pp. 1779–1792, 1995.
[17] K. Atkinson and W. Han, *Theoretical Numerical Analysis: A Functional Analysis Framework*, vol. 39 of *Texts in Applied Mathematics*, Springer, New York, NY, USA, 2nd edition, 2005.
[18] L. C. Evans, *Partial Differential Equations*, American Mathematical Society, Providence, RI, USA, 1998.
[19] A. N. Tikhonov, "Regularization of incorrectly posed problems," *Soviet Mathematical Doklady*, vol. 4, pp. 1624–1627, 1963.
[20] H. W. Engl, M. Hanke, and A. Neubauer, *Regularization of Inverse Problems*, Kluwer Academic, Dordrecht, The Netherlands, 1996.
[21] J. L. Lions, *Optimal Control of Systems Governed by Partial Differential Equations*, Springer, New York, NY, USA, 1971.
[22] P. Grisvard, *Elliptic Problems in Nonsmooth Domains*, Pitman, Boston, Mass, USA, 1985.
[23] S. C. Brenner and L. R. Scott, *The Mathematical Theory of Finite Element Methods*, Springer, New York, NY, USA, 2nd edition, 2002.
[24] P. G. Ciarlet, *The Finite Element Method for Elliptic Problems*, North Holland, Amsterdam, The Netherlands, 1978.

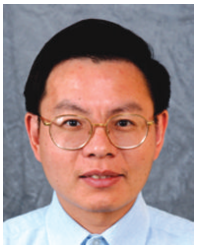

**Weimin Han** received his B.S. degree from Fudan University in 1983, M.S. degree from Chinese Academy of Sciences in 1986, and Ph.D. degree from University of Maryland in 1991, all in mathematics. He has been on the faculty of the Department of Mathematics, University of Iowa since 1991, as an Assistant Professor in 1991–1996, Associate Professor in 1996–1999, and Professor starting from 1999. His research interests are mathematical analysis and numerical approximations of problems arising in applications. He has authored/coauthored 7 books and more than 90 journal papers in numerical analysis, computational mechanics, and inverse problems.

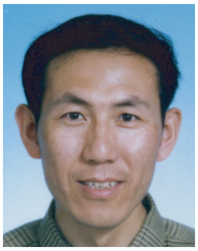

**Wenxiang Cong** received his Ph.D. degree in optical imaging from Beijing University of Science and Technology, Beijing, China, in 1998. Currently, he is a Research Scientist with Division of Biomedical Imaging, VT-WFU School of Biomedical Engineering and Sciences, Virginia Tech., Blacksburg, Virginia, USA. His research interests include biomedical imaging, optical tomography and bioluminescence tomography. He authored or coauthored 30 journal papers and 10 conference proceedings. He is a Member of the Optical Society of America.

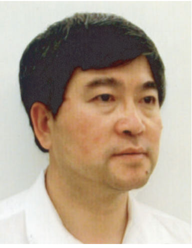

**Ge Wang** received the B.E. degree in electrical engineering from Xidian University, Xian, China, in 1982, the M.S. degree in remote sensing from Graduate School of Academia Sinica, Beijing, China, in 1985, and the M.S. and Ph.D. degrees in electrical and computer engineering from State University of New York, Buffalo, in 1991 and 1992. He was Instructor and Assistant Professor with Department of Electrical Engineering, Graduate School of Academia Sinica 1984–1988, Instructor and Assistant Professor with Mallinckrodt Institute of Radiology, Washington University, St. Louis, Mo, in 1992–1996. He was an Associate Professor with University of Iowa 1997–2002. Then, he was Professor with Departments of Radiology, Biomedical Engineering, Civil Engineering, and Mathematics, and Director of the Center for X-Ray and Optical Tomography, University of Iowa, until 2006. Currently, he is Samuel Reynolds Pritchard Professor of Engineering, and Director of Biomedical Imaging Division, VT-WFU School of Biomedical Engineering and Sciences, Virginia Tech. His interests include computed tomography, bioluminescence tomography, and systems biomedicine. He has authored or coauthored over 300 journal articles and conference papers, including the first paper on spiral/helical cone-beam CT and the first paper on bioluminescence tomography. He is the Editor-in-Chief for International Journal of Biomedical Imaging, and Associate Editor for IEEE Transactions on Medical Imaging and Medical Physics. He is an IEEE Fellow and an AIMBE Fellow. He is also recognized by a number of awards for academic achievements.

